# Hip Surgery Candidates: A Comparative Study of Hip Osteoarthritis and Prior Hip Fracture Patient Characteristics

**DOI:** 10.2174/1874325000802010079

**Published:** 2008-05-09

**Authors:** Ray Marks

**Affiliations:** Department of Health, Physical Education and Gerontological Studies and Services, City University of New York, York College and The Department of Health and Behavior Studies, Columbia University, Teachers College, New York, USA

**Keywords:** Hip, osteoarthritis, outcomes, surgery, trauma.

## Abstract

**Aim:**

To assess similarities and differences in patient-related characteristics before and after surgery for painful disabling hip osteoarthritis among elderly subgroups with and without a trauma history.

**Method:**

First, a cohort of 1000 hospitalized patients were assessed for trends in: perceived duration of the condition, pain intensity, functional performance ability, walking distance, body mass, and comorbidity characteristics among other factors. Then, the most salient of these patient-related characteristics were compared between 42 cases of hip osteoarthritis without a trauma history and 42 cases with a trauma history matched for age and gender, using medical records and standard data recording and analysis procedures.

**Results:**

Hip osteoarthritis cases with a prior hip fracture history had a longer duration of disability, and were more impaired functionally before surgery (p < 0.05) than those with no such history. They also had lower leg muscle strength and used more assistive devices.

**Conclusion:**

Patients undergoing hip replacement surgery for painful hip osteoarthritis who have a hip fracture history are likely to be more impaired and disabled than those with no such history.

## INTRODUCTION

Hip osteoarthritis is a common disorder affecting about 1.5% of United States adults. It is associated with increasing age, and often causes functional, social, and psychological disability, as well as financial difficulties. These problems arise as a result of the effects of the pain experienced and its chronicity. While the primary cause of hip osteoarthritis is often unknown, the disease commonly occurs secondarily to hip fracture injuries, as a result of trauma and surgery for this condition [1] among other factors. However, because the end-point of this disease is commonly quite similar, regardless of disease cause, most patients will receive similar treatments to alleviate their disability. That is, most will undergo some form of total hip replacement surgery [2]. In addition to being a somewhat standardized technique, this surgical approach, introduced in the 1960s to alleviate hip osteoarthritis disability and restore function, is often followed by standard post-operative management procedures. However, while generally successful in achieving its goal, this therapeutic approach does not take into account the fact that variations in the disease prevail and evidence that variations in outcome exist [3]. These variations [3] may be due to patient-related, rather than technological factors [4] and therefore amenable to examination and possible remediation. In particular, the literature suggests that the hip fracture patient who develops secondary hip osteoarthritis following failure of their original surgery and fixation, may be more impaired both prior to and following surgery to replace the diseased hip.

One reason for this is that patients who have a hip fracture history may present with poor pre-operative walking capacity and deficits in hip flexion range of motion. Research shows that the presence of one or both of these functional problems makes it less likely that these patients will achieve the same outcome with regard to walking and motion than those with better pre-operative functional ability [5].

While secondary arthritis of the hip can stem from other causes, such as infection, and such cases may similarly have less favorable outcomes than those with primary hip osteoarthritis, one very common problem among the rapidly aging population is the number of cases who are predicted to fall and fracture their hip. Because of the high percentage of the aging population expected to have primary hip arthritis, along with many of the approximately 340,000 hip fractures cases that occur each year among American adults ages 65 yrs or older at an estimated cost of $ 2.9 billion, it seemed especially relevant to examine whether the patient with a past hip fracture history who develops secondary hip osteoarthritis that requires total hip arthroplasty surgery may need more intense pre and/or post-operative rehabilitation as a preferred tertiary preventive strategy. That is, if hip replacement surgery is required for aging adults who have previously sustained a hip fracture, is more targeted and more intense pre- and post-operative rehabilitation required for this cohort as indicated by Kessler [3].

If so, even though small numerically speaking in relationship to non-traumatic cases requiring hip replacement surgery, since the literature suggests the disability recorded for past hip fracture patients undergoing hip replacement surgery for secondary hip osteoarthritis pre-and post-operatively is considerable, as are the costs involved, it seemed a study to address this question would be valuable. Moreover, since patients with a prior trauma history, especially those sustaining a hip fracture may be at risk for secondary hip arthroplasty surgery, and would potentially present a greater challenge to the rehabilitation team, it was felt important to examine this issue. Consequently, the overarching goal of this research was to examine if the pre-surgical clinical presentation and short-term functional outcomes would differ in any way between patient subgroups 60 years and older undergoing hip joint arthroplasty for end-stage hip osteoarthritis in the same hospital setting. This line of investigation specifically pertains to the goal of implementing optimal clinical pathways and improved tertiary preventive guidelines for this group. It also pertains to the need that has been demonstrated to examine whether osteoarthritis outcomes and thus patient and societal costs can be improved through careful sub-group analyses. Although some work has been done to examine functional status and pain among adults with hip osteoarthritis undergoing hip surgery [4], very little has been done to examine if the disease manifests differentially for selected subgroups. Since research that exists is equivocal as to whether pain and function deteriorate or not over the course of time, the reason for this discrepancy may be due to a failure to carefully examine clinical and other factors known to be related to functional status. One such factor is prior trauma, especially that resulting in a hip fracture, which may heighten the disability and its duration and severity.

To address these aims, and to ascertain the extent to which trauma might be implicated in hip osteoarthritis disease progression, we first estimated the percentage of such cases among 1,000 consecutive hip arthroplasty patients awaiting surgery. We then sought to establish trends among patients with one of four categories of trauma history: a first hip fracture; a history of an old hip fracture; a history of fractures other than the hip; those with a falls related history but no fracture, and the presence of any trend or variation in functional presentation at baseline and immediately after surgery. Third, a sub-group analysis of past hip fracture injury and age-and gender matched atraumatic hip osteoarthritis cases undergoing the same surgery was conducted.

## METHODS AND PROCEDURES

As part of a comparative strength testing related study that received ethics approval, the present analysis involved an initial cross-sectional analysis conducted retrospectively from the historical records of 1,000 cases of hip osteoarthritis replacement surgical cases with/without a trauma history over a sixth month period in the same venue, a renowned orthopedic center serving a large urban community. Selected features at baseline prior to surgery and immediately after surgery that were recorded on each patient’s chart were examined to describe or uncover any trends or variations among these subgroups as follows: 

 Evidence of pre-operative assistive device use, categorized as Yes or No.

 Self-reported pre-surgical maximal walking distance in numbers of blocks.

 Self-reported pre-surgical stair climbing ability, categorized as: Reciprocal or normal; Non-reciprocal; Unable.

 Post-operative walking distance on Days 1 and 3 of the post-surgical period, as recorded by the therapist on the patients chart in feet.

 Age, medical comorbidity presence and number (excluding psychiatric or behavioral problems), evidence of any leg strength deficit as recorded using standard manual muscle tests ranging from 1-5, where 5 is Normal strength and 1 is Impaired Strength, weakness for the key muscles of affected leg categorized as Yes or No (Grade 4 or lower), discharge destination (categorized as Home or Rehabilitation Center) and length of stay in hospital (Days).

Based on trends observed when analyzing these data, and to control for potentially confounding effects of age, gender and acute versus chronic trauma history on the disease process, similar data from a sample of 84 of these patients selected at random and who were 60 years or older with either a non-traumatic or a past hip fracture history were examined (that is, almost all cases in the original cohort of 1000 cases with a past hip fracture history who were 60 years or older). More specifically, body mass indices (the ratio of the patient’s weight divided by the square of their height-w.h^-2^), the presence of any medical comorbidity, the duration of the condition, pain (as recorded on a 1-4 Likert scale ranging from Mild to Severe), baseline hip flexion and internal rotation range of motion (in degrees as recorded by the physician), pre-surgical walking status, leg strength, and short-term functional recovery status as recorded on the charts by post-surgical walking distance were compared across these two sub-groups groups.

All data were extracted systematically from the patients’ records by a single recorder and entered into a spreadsheet. Thereafter, descriptive and inferential statistics including 2-sided Pearson Chi-Square, Cross-tabulations, Levene’s Test for Equality of Variances T-Tests, Analysis of Variance and selected correlational analyses were generated using SPSS version 12.00. It was assumed that the data adequately represented trends in the above variables, as systematic charting practices and validated recording tools were used throughout. Excluded from the analyses were subjects who had a rheumatoid arthritis history; were suffering from hearing impairments, dementia or related cognitive problems, those who had been discharged before their data could be collected, those whose records were not available at the time of analysis, and those who were permanently disabled.

## RESULTS

The large cohort was constituted by 1000 cases diagnosed as having clinical and radiographic hip osteoarthritis requiring replacement surgery, ages 26-93, mean age 65±13.4 years. Thirty percent were younger than 60 years of age and 60% were females. The average age of selected categories of patients among the large cohort showed variation, particularly between those with primary versus those with secondary hip osteoarthritis (65.0 years versus 70.25 years). That is, those with secondary hip osteoarthritis were older on average than those with primary hip osteoarthritis.

Figs. (**1**,**2**) illustrate the percentage of cases that had a trauma history, and the breakdown of these trauma types. As shown, more patients were classified as having a non-traumatic disease associated history, and of the 13% of cases who had a hip fracture history, 9% reported a past hip fracture history.

**Fig. (3):**
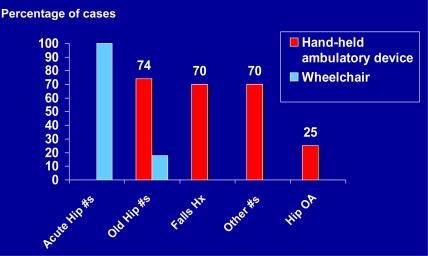
Frequency distribution of assistive device usage as recorded preoperatively and categorized by diagnosis for 1000 cases.

In terms of a proxy for disability levels at baseline prior to surgery, Fig. (**3**) displays the trends observed in the use of assistive devices among the larger cohort. As shown, the use of mobility devices, which may denote levels of independence and life quality, was reported more frequently by those cases with a traumatic history. Additionally, more patients in the post-traumatic arthritis group reported being wheelchair bound than those in the non traumatic group (Fig. **3**). As confirmed by the sub-group analyses, pre-surgical device usage was more prevalent among those patients with a trauma history than those with no trauma history (p=.023). As displayed in Table **1**, the demographics of the two sub-groups showed even though subjects were matched for age, numbers of affected joints and gender (females=25 subjects; males=17 subjects in both groups), several features distinguished the two groups at baseline, in particular, years of disability.

**Fig. (4):**
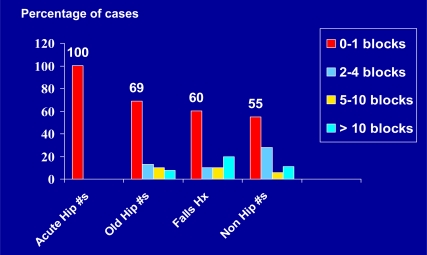
Frequency distribution of numbers of blocks patients could complete prior to surgery according to self-reports across 1000 cases.

In terms of maximal numbers of blocks patients said they could negotiate prior to surgery, those with a hip fracture history performed less well than those who had fractures of other joints or had experienced past falls (Fig. **4**).

In line with the finding that pre-surgical walking distances were significantly correlated with post surgery Day 1 (r=285; p=.020) and Day 3 walking distances (r=356; p=.090), Fig. (**5**) shows that when a comparison was done to establish estimates of walking ability before surgery for the 1000 hip osteoarthritis cases, the post-traumatic hip arthritis cases appeared to exhibit a trend towards poorer pre-surgical ambulatory function than those with no such history.

Similarly, even when age, and numbers of patients were controlled for in the analysis, in terms of disability levels, the average number of blocks reported for the traumatic sub-group of 1.76 + 2.84 was generally lower than that of 2.85 + 2.89 recorded for the non-traumatic sub-group (p=.099).

In terms of pre-surgical stair climbing ability, Fig. (**6**) shows the extent of this function was more limited for those with a hip fracture history when compared to their counterparts with no trauma history. As confirmed by a Chi-Square analysis applied to the 42 trauma and 42 atraumatic hip surgery candidates, pre-surgical stair climbing ability was generally different for normal reciprocating or non-reciprocating ability, and inability to negotiate stairs between these two hip osteoarthritis sub-groups. Those with a history of past hip fractures showed a higher rate of being unable to climb stairs and a lower rate of being able to carry out normal reciprocal stair climbing activities (p=.001).

**Fig. (8):**
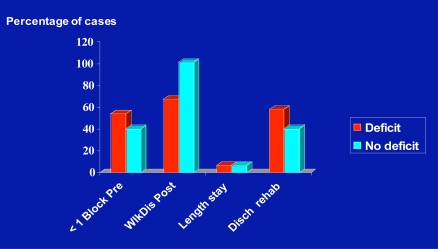
Graphic representation of pre-surgical and functional outcomes categorized by the presence or absence of a pre-surgical leg strength deficit on operative side.

As shown in Fig. (**7**) for the larger cohort comparisons, there was generally a higher rate of functional recovery for the cases without a trauma history than those with a hip fracture history in the post-operative period. As well, those with an acute fracture appeared to recover more slowly, than those with a past hip fracture history. This could reflect the finding of the presence of a higher frequency of operative leg muscle weakness at baseline for the traumatic hip fracture surgery cases and better leg strength for those with no trauma history who had also had higher levels of pre-surgical function (Fig. **8**). As well, among the sub-groups studied, the percentage of cases presenting with weakness among the cohort of past hip fracture patients of 23/38 tended to be higher than the rate of weakness among the atraumatic cases of 15/38 (p=.090). Other findings were that among the correlations assessed, hip flexion joint range of motion and pain were negatively correlated (r=-.309; p < 0.05), and the leg strength and hip flexion and internal rotation joint range of motion data, which were significantly correlated (r=.477; p=.000) were positively associated with the Day 1 and Day 3 walking scores of 4.63±8.33 versus 7.50+11.79 ft, and 64.97±74.11 versus 67.50±64.42 ft, respectively. There was also a trend towards being discharged to a rehabilitation center, rather than home among patients with identified muscle weakness at baseline (p=.052), but discharge destination, where 50% or more of cases from each group went to a rehabilitation center and did not go directly home did not differ among groups (p=0.302). Correlations related to Day 1 and Day 3 walking distances, which were significantly associated (r=.710; p < 0.05) are shown in Table **2**. The impact of back problems that can affect the elderly was negligible though and the rates of back pain or having a back surgery history were comparable among the groups (5.5% for primary cases versus 5.0% for the secondary hip osteoarthritis cases).

Table **3** shows that those secondary hip osteoarthritis cases with high numbers of comorbid disease or higher body mass indices are more likely to exhibit leg strength deficits than those who are within normal body weight ranges with fewer comorbid conditions.

## DISCUSSION

Hip joint osteoarthritis - often associated most closely with aging - and producing a significant burden of the disability experienced by older people, is an often overlooked potentially preventable or remediable public health problem [4]. Moreover, even though the extent of this disability may vary, very little research has been conducted to examine whether different categories of this condition require different levels of clinical intervention in order to maximize an individual’s well-being. That is, there very little data to support the view that certain clinical features, rather than technological explanations, may partially explain the lack of uniformity in outcomes reported in the literature for people diagnosed as having debilitating hip joint osteoarthritis requiring hip replacement, who are often treated quite similarly after surgery.

Given the costs of hip osteoarthritis both to society and to the individual, an examination of whether certain hip osteoarthritis sub-groups may recover more slowly than others after similar surgery and rehabilitation, if salient differences in baseline predictors of function are not identified before surgery seemed indicated [6]. In particular, even though they might constitute only a small percentage of hip replacement cases, it was felt the disability experienced by those patients with a past hip fracture history undergoing such surgery would be especially high because the condition itself may pose increased intra-operative difficulties [7]. Because this has not been well studied, despite the potential implications of this information in efforts to reduce the overall disease burden, this present study aimed to explore whether a hip osteoarthritis patient's pre-surgical history is uniform regardless of disease cause and if not if this was likely impact short-term post-operative outcome in some way. The specific short-term goal was to ascertain if it may be helpful to recommend identification of-and more detailed pre-surgical assessments be conducted for this patient group. The long-term goal was to improve opportunities for maximizing the post-surgical outcomes for older people with debilitating hip osteoarthritis, given the increasing aging population.

After first establishing the extent to which secondary hip osteoarthritis due to trauma prevailed among a large representative group, several disability indicators were assessed and compared among selected sub-groups to establish trends in these data. Thereafter, similar trends when controlling for age, numbers of affected joints and gender were sought.

In terms of overall findings, similar to Brown *et al*. [6], approximately 13% of the overall prevalence of symptomatic end-stage hip osteoarthritis was due to trauma, especially hip fractures due to falls. In addition, those with a traumatic history were more likely to be impaired both before and after surgery than those with no trauma history. In particular, in addition to disability as measured by blocks walked, and hip flexion range of motion, the data revealed clinical differences in pain experience, and hip internal rotation limitations of movement and comorbidity distribution suggestive of somewhat different clinical representations of the generic condition of hip osteoarthritis. Given that high numbers of older people are likely to be affected in the future by post-traumatic hip osteoarthritis that will require hip replacement surgery, the ability to lessen this burden through a comprehensive pre-surgical analysis that allows for more tailored interventions and predictions of need will be potentially beneficial. This argument is further strengthened given that the subset of patients studied were well matched for age, gender, numbers of affected joints.

For example, in the present study, the presence of a marked deficit in the estimates of the patient's hip flexion range of motion, if one compares this to the normal expected range of motion of this joint, which was similar to that described by Roder [5], was correlated with the physician-based estimates of hip internal rotation joint range of motion, a measure negatively correlated with the patient’s self-reported overall pain intensity levels (r=-.309; p=.016). The hip flexion joint range of motion measure was also positively associated with Day 1 walking distance (r=.233; p=.056) and a similar trend was observed for the association between these range of motion measures and the patient's Day 3 post-surgical walking distances as well (r=.250; p=.076).

The measurement of hip flexion joint range of motion currently reported was one assessed uniformly across all patients to provide an overall estimate of the muscular power of the affected lower leg. Thus, the present results revealed that lower levels of leg power, potentially predict pain and functional recovery, two issues of key import in the context of attempts to maximize outcomes for people requiring hip surgery for disabling hip osteoarthritis. This observation also accords with recent research emphasizing the importance of muscle strength in the performance of physical tasks [8,9] and may partly explain the greater pre-surgical difficulties reported on stair climbing by the past hip fracture sub-group. Device use, which was also higher in this group, and could be viewed as an indicator of the greater impairment experienced by this group who had long histories of lower leg dysfunction, may also represent the presence of poor leg muscle strength and/or endurance.

The present data also revealed that pain levels did not explain the pre-surgical performance ability of the hip osteoarthritis patient. However, in addition to evidence of hip muscle weakness on the affected side, there was strong evidence of knee extensor muscle weakness among many of the patients, which could heighten disability, and the ability to function physically. This latter observation is consistent with the view of Rasch *et al*. [9] that the major muscles functioning around the hip and knee may show substantial loss of strength and mass in people with hip joint osteoarthritis and this could contribute to the reduced ambulatory capacity of these patients. It could also exert a powerful influence on falls that lead to injuries [10] and second fractures, other fractures (which occurred at higher rates in those who were 60 years and older and required hip replacement surgery as shown in Fig. (**2**)) or poor post-surgical outcomes as outlined in Fig. (**8**).

The greater number of years since the onset of their hip problems and/or the presence of one or more comorbid conditions may explain the marked deficits in function at baseline of those with a trauma history and a slower recovery rate even after receiving state of the art surgery. It is becoming increasingly clear though that pre-existing hip and knee strength potentially predicts or explains why not all hip replacement surgical patients are ambulatory to the same degree after surgery to relieve pain and repair damaged joint structures. Verification of the importance of muscle strength in predicting functional outcomes could be helpful clinically, given that muscle strength can be increased, regardless of age. However, to establish this proposition requires further study of larger samples, because in the current instant not all patients could undertake the walking activity and thus the sample size for these Day 1 and 3 data may have been too low to identify clinically important functional differences among these groups. Indeed, the available data may simply have represented those who already had more favorable, rather than less favorable outcomes. Other limitations of the present study are that it was cross-sectional to a large degree and employed self-reports.

In terms of hip fracture cases that may be requiring hip replacement surgery for secondary osteoarthritis, the present findings are consistent though with those of Brown *et al*. [6]. That is, it seems reasonable to suggest that a similar percentage of hip osteoarthritis cases presenting for surgery in other venues are likely to have this type of history. Moreover, although this present analysis was limited to one hospital location and one cohort over a restricted time period, it seems it is clear the patient with secondary hip osteoarthritis may require more specific or prolonged pre-surgical and post-surgical intervention to maximize the utility of the surgical intervention. In addition, it is likely that this group has poorer overall baseline function, because they have suffered from their disability for a significantly higher number of years, along with experiencing failure of their original internal fixation. As well, given that other risk factors for poor function differed among the sub-groups studied, it seems plausible to suggest the two sub-groups presently studied may represent different disease pathways, one being due to the presence of obesity and comorbid diseases, the other due to a more active lifestyle-that exposes people to the risk of fracture more readily. Perhaps the former develop more pain sooner and different restrictions on their mobility pattern that bring them to surgery at an earlier point in time, where they are less debilitated in general and can respond more ably or rapidly to the surgical procedures.

While other limitations to this research include, the use of retrospective data, and short-term hospital based outcomes only, these present observations, which encompass a representative sample of patients who attend the hospital each year for reconstructive surgery of the hip, are relevant in our view because research shows that age alone does not affect the outcome of joint replacement [11]. Research also shows that with increasing life expectancy, the goal of improving life quality for all, cannot be achieved if interventions are standardized and delivered in a generic manner to all patients given their varied degrees of health [7]. Accounting for a patient’s unique history in planning for their rehabilitation and recovery, will undoubtedly enable clinicians to design more optimal rehabilitation strategies.

One of the *Healthy People* *2010* goals, an American preventive health initiative, is to maximize number of years of healthy life, and the quality of that life. However, it appears that some cases of hip osteoarthritis are experiencing a greater demise in their life quality than others, and may not always receive the help needed to maximize their well being due to uniform surgical procedures and rehabilitation processes that are not necessarily tailored to the specific candidate because it is assumed the disease process is somewhat uniform. This concurs with the observation of Williams *et al*. [12] that comorbidity care is often poorly coordinated prior to surgery, during the acute care stay and following surgery and primarily entails prescribed medicines, even though its presence is associated with poorer outcomes, especially among hip fracture cases [13]. Attention to the aging hip osteoarthritis patient is also indicated, because preventable comorbid diseases that can occur as a consequence of immobility and pain are associated with greater post surgical functional limitations [14], and lower walking distances for up to 3 days after surgery if compared to those with no comorbidity.

Moreover, patient satisfaction, a key indicator of outcome is likely to be heightened for adults over 60 years of age with secondary hip osteoarthritis due to a hip fracture if these patients are carefully examined at baseline to assess their physical and functional status and the implications of these features can be taken into account in planning the post-intervention rehabilitation process. This is because, if patients as well as their physicians and rehabilitation team have a mutual understanding of the realities of the expected surgical outcomes, not only can more tailored collaborative strategies be implemented, but if the prognosis is clarified for the patient, unrealistic outcome expectations may be averted. Measurable predictors of intervention outcome variations are likely to be: age, lower leg strength, years of disability, walking distance, device usage, stair climbing ability, comorbid health conditions, pain intensity and hip joint range of joint motion.

In this respect, it is the author’s view that future research to further examine some of the aforementioned findings and premises using more sophisticated outcome measures and a more protracted study period will be helpful in advancing the outlook for older people with a hip fracture and/or hip osteoarthritis diagnoses. It may also be helpful to examine if one can prevent the development of some cases of secondary arthritis by careful intervention and follow-up of the acute hip fracture patient before the development of secondary osteoarthritis. As outlined by van Dijk [15] and Kessler *et al*. [3], the identification of risk as well as protective factors against severe disabling hip osteoarthritis appears to warrant more attention, as does the classification of the complex interaction of biomechanical factors, clinical factors, and treatment related factors that influence hip osteoarthritis outcomes.

## Figures and Tables

**Fig. (1):**
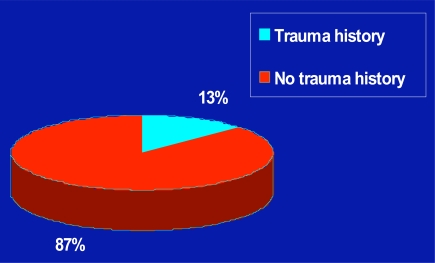
Pie chart showing the distribution of the etiological characteristics of 1000 patients undergoing total hip replacement surgery by trauma history.

**Fig. (2):**
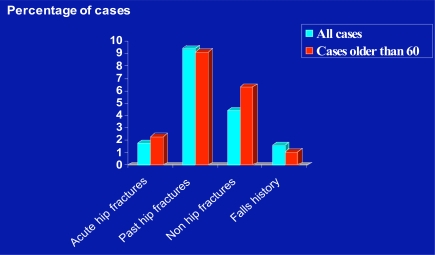
Bar chart showing the frequency of first or prior hip fractures versus frequency of hip surgeries for other reasons among a cohort of 1000 cases ranging in age from 26-90 years versus the cohort represented only by those older than 60 years of age.

**Fig. (5):**
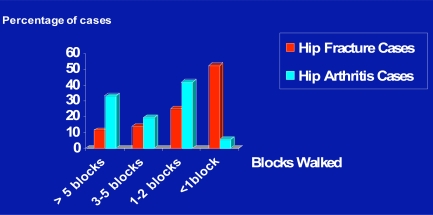
Maximal pre-surgical walking distances categorized by numbers of blocks for hip fracture cases with secondary hip osteoarthritis versus hip osteoarthritis cases.

**Fig. (6):**
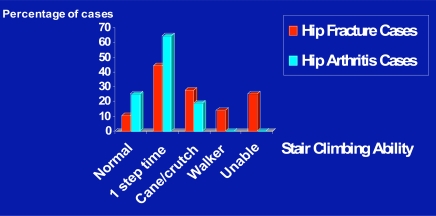
Overview of the trends exhibited in pre-surgical stair-climbing ability among the past hip fracture and primary hip osteoarthritis patients.

**Fig. (7):**
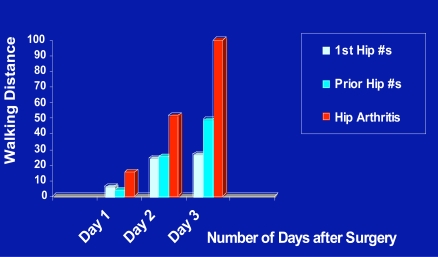
Graphic depicting trends in post-operative walking distances attained between post-operative days 1-3 by diagnostic category.

**Table 1.** Characteristics of 42 Hip Osteoarthritis Cases with a Trauma History and 42 With No Trauma History, Ages 60 Years or Older, As Recorded Prior to Surgery for Debilitating Osteoarthritis Showing Significant Differences in Years of Disability and Trends Towards Greater Physical Limitations Among the Traumatic Group

Subgroup			
Variable	Trauma	No Trauma	p-Value
Age (years)	74.05±8.96	72.48±7.52	.386
BMI (w.h^-2^)	25.3±4.8	26.6±5.3	.291
Comorbid #	1.07±.89	1.13±.92	.233
Yrs disability	8.75±10.00	3.11±3.03	.002*
Blocks walked	1.76±2.83	2.85±2.88	.099
Hip flex (deg)	73.86±30.3	84.58±22.43	.082
Int rot (deg)	6.47±6.8	5.05±6.45	.364
Pain (1-4 scale)	2.72±1.01	3.10±.95	.119
**Number Reporting:**			
Stair climbing problems	30	26	
No stair climbing problems	6	10	.005*
Device use	30	22	.023*
# leg weakness	23	15	
# leg no weakness	14	24	.090
Bilateral disease	8	4	.212

**Statistical tests:**

Levene's Independent samples 2-tailed t test.

Pearson Chi-Square.

**Table 2.** Selected Correlations Among the Key Variables Studied Depicting Potentially Important Predictors of Short-Term Post-Surgical Outcomes for People with Hip Osteoarthritis 60 Years of Age or Older

Distances Walked Post-Surgery		
	Day 1	Day 3
Age (years)	-.146	-.227
# comorbidities	-.244*	-.420**
Blocks walked	.285*	.356**
Hip Flexion (degrees)	.233	.250
Hip Internal Rotation (degrees)	-.016	.221
Pain (1-4 Likert scale)	-.065	-.081
Day 1 walking distance (feet)		.710**

** Pearson correlation is significant at the 0.01 level (2-tailed).

* Pearson correlation is significant at the 0.05 level (2-tailed).

**Table 3.** Characteristics of Patients with Secondary Hip Osteoarthritis by Strength Deficit of the Affected Leg.

	Age (Years)	BMI (w.h^-2^)	Comorbidity #	Gender
With deficit	74.68	26.2	3.5	35% M
				65% F
No deficit	74.4	21.8	1.35	40% M
				60% F

BMI=body mass index; F=female; M=male; #=number.
